# Randomized blinded pilot trial of corticosteroid after mild-moderate anaphylaxis to prevent recurrence

**DOI:** 10.1097/MD.0000000000043600

**Published:** 2025-08-01

**Authors:** Rafah Al Sayyed, Khalid Alansari

**Affiliations:** a Department of Pediatric Medicine, Hamad Medical Corporation, Doha, Qatar; b Department of Pediatric Emergency Medicine, Sidra Medicine, Doha, Qatar; c Weill Cornell Medical College, Doha, Qatar; d Qatar University Medical College, Doha, Qatar.

**Keywords:** anaphylactoid, anaphylaxis, corticosteroid, dexamethasone, emergency, epinephrine

## Abstract

**Background:**

Corticosteroid appears associated with shortened hospital stay for anaphylaxis in hospital-admitted children but whether Emergency Department (ED)-discharged patients will benefit is uncertain. Unnecessary routine corticosteroid use can have adverse effects.

**Methods:**

Pilot blinded randomized trial of children 3 months to 14 years presenting with anaphylaxis, comparing ED predischarge 0.3 mg/kg oral dexamethasone solution, 1 mg/mL, maximum 10 mg, or an equal volume of blinded identical placebo, to determine if revisit for recurrence to any health care facility within 7 days would be reduced.

**Results:**

Covid-19 resource reallocation resulted in early recruitment suspension. One hundred fifty two patients were randomized, 68 dexamethasone and 75 placebo recipients analyzed. Only 5/75 (6.7%) of placebo recipients required returning, compared to 3/68 (4.4%) for dexamethasone, absolute risk reduction 2.3%, number needed to treat 44 (95% confidence interval, 12 to an infinite), *P* = .7.

**Conclusion:**

Uncommon recurrence and high number needed to treat after ED anaphylaxis treatment suggests against routine ED predischarge corticosteroid without a clinical rationale.

**Key points**

• Use of steroid to treat mild-moderate anaphylaxis is based on experience and preference with no prospective clinical trial to support or discourage its use.
• Uncommon recurrence and high NNT after ED anaphylaxis treatment suggests against routine ED predischarge corticosteroid without a clinical rationale.

## 1. Introduction

While corticosteroid treatment, with epinephrine and antihistamine, appears associated with shortened hospital stay for children admitted with severe anaphylaxis^[1]^ whether there is benefit of corticosteroid for Emergency Department (ED)-discharged patients for preventing anaphylactoid (recurrence and revisit within several days after discharge) is uncertain. When 66% of 5052 children were ED-discharged having received corticosteroid, 5% of steroid recipients and non-recipients returned to the ED within 3 days.^[2]^ Both North American and European guidelines identify corticosteroids as optional therapy for discharged patients.^[3,4]^ Adverse effects may accompany frequently administering corticosteroid for mild-moderate anaphylaxis^[5]^ because some patients present frequently and may have coexisting comorbid conditions. Anaphylaxis is not rare^[6]^; by September 2016, our ED had treated 171 cases of anaphylaxis in the preceding 12 months, often providing corticosteroid.

## 2. Materials and methods

### 2.1. Setting and participants

The study was conducted between June 2018 and February 2024 in the short stay unit of the Pediatric Emergency Center (PEC) of Hamad General Hospital, one of the main pediatric emergency facilities in the State of Qatar. PEC also contains 42 beds in a short stay infirmary unit. Patients admitted to the unit are assessed at least every 6 hours by a pediatrician to determine medical readiness for discharge. The length of stay in the unit for anaphylaxis ranged from 2 to 50 hours. Written informed consent was sought from one of the parents or legal guardians and assent for children more than 7 years of age for consecutive eligible patients was obtained for all participating patients. The study was approved by the hospital institutional review board and registered (clinicaltrials.gov, ID #NCT03523221).

Children 3 months to 14 years presenting to the unit for the treatment of mild to moderate anaphylaxis were eligible for the study. Mild-moderate anaphylactic was defined as an acute allergic reaction with at least 2 systems involvement: skin, cardiovascular, respiratory or gastrointestinal, and without presence of hypotension, laryngeal spasm, loss of consciousness, or neurological compromise.

Patients were excluded from the study if they had 1 or more of the following characteristics: severe anaphylaxis, moderate to severe asthma and routinely taking asthma medication, has immunological disease, or on corticosteroid treatment.

### 2.2. Study procedures

This was a double blinded randomized placebo-controlled study. Patients were examined on presentation and those diagnosed with anaphylaxis were admitted to the short stay infirmary unit. Those with acute anaphylaxis were assessed for study eligibility within 15 minutes of the initial physician assessment. Patients for whom written informed consent was obtained received our standard treatment, epinephrine and H1-antihistamine, with education related to the cause of anaphylaxis and avoiding future episodes. Study medication was administered within 30 minutes of patient arrival to short stay unit. A previously computer-generated list of random numbers generated by the study statistician was used by the enrolling physicians in consecutive order to identify a sealed envelope, which was accessed and unsealed only by the preparing pharmacist who was blinded to patient identity. The envelopes contained one of the 2 study medications. A study pharmacist prepared the study medication, dexamethasone syrup at 0.3 mg/kg, 1 mg/mL, maximum dose of 10 mg or placebo with the same volume, color and taste. Study allocation concealment was ensured throughout enrollment.

When patient was determined to be ready for discharge as per the treating physician discretion, he/she was sent home to complete 5 days course of oral cetirizine. Study nurse follow-up by telephone was mandatory daily for 1 week after discharge. The patient could return to the PEC earlier.

### 2.3. Outcome measures

Need for revisit or readmission for anaphylactoid reaction to any health care facility within 7 days after discharge.

### 2.4. Sample size and analysis

We used a retrospective 30 charts review for revisits within 7 days to arrive at a 35% revisit rate for placebo group. Expecting 50% improvement for dexamethasone arm, a 2-sided significance level of 0.05 with power of 80% resulted in a sample size estimate of 90 patients per group, to compensate for drop out 212 were planned to be recruited altogether percentage and mean with standard deviation. Quantitative variable means between the 2 treatment groups were analyzed by unpaired t and Wilcoxon rank sum tests. Associations between qualitative and categorical variables were assessed by chi-square testing, with the continuity correction for smaller cell frequencies. Statistical analyses were performed using SPSS (version 19.0r, IBM SPSS Statistics, IBM Corporation, Armonk).

## 3. Results

**Figure 1.**
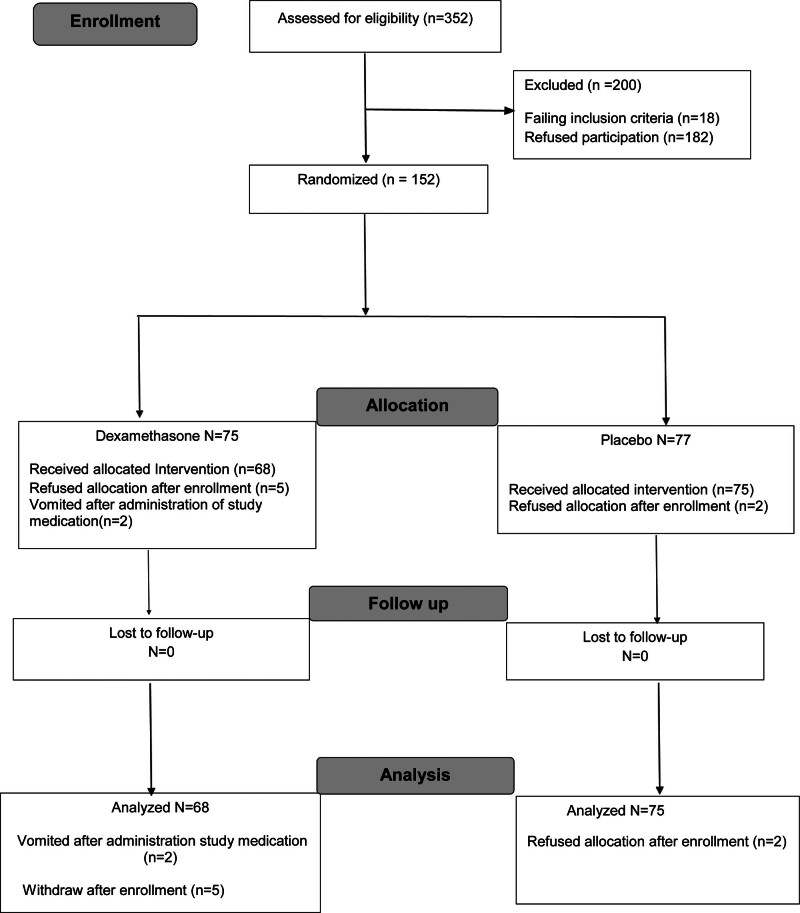
Consort form of patient flow.

Our study started with institutional approval April 2018 and the first patient was enrolled in June 2018. When Covid-19 spread to Qatar early in 2020, resources for this and other ongoing clinical trials were redirected. Over time, there was attrition of personnel and other institutional study resources and recruitment was terminated, the data base locked, and treatment unblinded September 2024. Three hundred fifty two patients were assessed for eligibility, 152 randomized, 75 to dexamethasone and 77 to placebo. 68 dexamethasone recipients were analyzed, since 2 vomited medication and 5 withdrew. Seventy five placebo recipients were analyzed after 2 withdrew. No treated patients were lost to follow-up. Patient flow is seen in Figure 1. Patient characteristics are seen in Table 1.

**Table 1:** Baseline characteristics of children with anaphylaxis.

	Dexamethasone (N = 68)	Placebo (N = 75)
Age, yr, mean (SD)	4.11 (3.68)	4.41 (4.05)
Weight, kg, mean (SD)	18.72 (13.42)	19.64 (14.31)
Sex, n%		
Male	44 (64.7%)	43 (57.3%)
Female	24 (35.3%)	32 (42.7%)
Previous history of anaphylaxis, n%	18 (26.5%)	29 (38.7%)
Previous history of food allergy, n%	22 (32.4%)	27 (36.0%)
Previous history of asthma, n%	4 (5.9%)	3 (4.0%)
Previous history of eczema, n%	22 (32.4%)	26 (34.7%)
Family history of allergy, n%	18 (26.5%)	19 (25.3%)
Anaphylaxis involved system		
Mucocutaneous, n%	56 (82.4%)	58 (77.3%)
Respiratory, n%	43 (63.2%)	43 (57.3%)
Gastro intestinal system, n%	29 (42.6%)	39 (52.0%)
Cardiovascular system, n%	24 (35.3%)	27 (36.0%)
Neurological system, n%	7 (10.3%)	9 (12.0%)
2 Systems involved, n%	28 (41.1%)	31 (41.3%)
More than 2 systems involved, n%	40 (58.8%)	44 (58.6%)
Causes of anaphylaxis		
Medication/vaccination, n%	1 (1.5%)	5 (6.7%)
Food, n%	59 (86.7%)	58 (77.3%)
Nuts	19 (27.9%	13 (17.3%)
Egg	8 (11.8%)	12 (16.0%)
Dairy products	6 (8.8%)	22 (29.3%)
Fish	1 (1.5%)	1 (1.3%)
Others	25 (36.7%)	10 (13.3%)
Insect bites, n%	7 (10.3%)	12 (16.0%)
Exercises/weather changes, n%	0	0
Unknown, n%	1 (1.5%)	0
Needed NS bolus, n%	0	0
Epinephrine received before arrival at home, n%	6 (8.8%)	3 (4.0%)
Epinephrine received after arrival, n%	62 (91.2%)	72 (96.0%)

NS = normal saline, SD = standard deviation.

Our projected 1-week placebo return rate of 35% proved to be an overestimate; 5 of the 75 returned, 6.7% (95% confidence interval [CI], 2.2–14.9%). Other study results are presented in Table 2. Among dexamethasone recipients, the 7-day return rate was 3 of 68, 4.4% (95% CI, 0.9–12.4%). While the relative risk reduction, not significant, was 33.8% (95% CI, 87.3% to an increased risk of 208%), the absolute risk reduction was only 2.3%, yielding an estimated number needed to treat to prevent 1 revisit during 7 days of 44 patients (95% CI, 12 to an infinite number). Subgroup analysis based on anaphylaxis severity, cause of anaphylaxis, and involved systems was performed but yielded no significance.

**Table 2:** Primary and secondary efficacy of dexamethasone versus placebo.

	Dexamethasone (N = 68)	Placebo (N = 75)	Absolute risk reduction (95% CI), NNT*
Primary outcome			
Revisit to any healthcare facility with anaphylactoid reaction within 1 wk of follow-up, n%	3 (4.4%)	5 (6.7%)	2.3% (−5.8 to 8.3%) NNT 44, *P* = .7
Secondary outcome			
Readmission to any healthcare facility because of anaphylactoid reaction within 1 wk of follow-up, n%	0	2 (2.7%)	2.7% (−1.8 to 2.7%) NNT 38, *P* = .5
Need for hospital admission, n%	0	2 (2.7%)	2.7% (−1.8 to 2.7%) NNT 38, *P* = .5
Need for ICU admission, n%	0	0	–

CI = confidence interval, ICU = intensive care unit , NNT = number needed to treat.

* NNT is number needed to treat for benefit.

There were no study drug-specific adverse events reported during hospital visit or in follow-up except for 2 patients who vomited after oral dexamethasone.

## 4. Discussion

To our knowledge this is the first randomized clinical trial to test the effect of steroids in decreasing anaphylactoid reaction in pediatric patients treated for mild-moderate anaphylaxis

This pilot study was undersized by 37 patients to define the absolute benefit of dexamethasone in preventing need for return. However, our rate of return in the placebo group, 6.7%, is consistent with prior estimates. But the estimated number needed to treat of 44, lower limit of 12, suggests to us that dexamethasone treatment is not routinely warranted. There may be some ED-discharge patients with a history of recurrent anaphylactoid reactions in whom dexamethasone is perceived to be warranted to try to avoid this complication.

This pilot trial was limited by small sample size, caused by pre-study over estimation of return visit, and low frequency of need to return post-analysis, although a frequency consistent with other reports from elsewhere. Study strengths include blinding and a dexamethasone dosage intended to provide a robust test of the drug’s effect.

We conclude that dexamethasone treatment of ED-discharged anaphylaxis children aged 3 months to 14 years should not be routine, but rather restricted to those patients for whom there is a sound clinical rationale, because the proportion of patients likely to be spared return within 7 days if all are treated is quite small.

## Acknowledgments

We extend our appreciation to all individuals who directly or indirectly contributed to this project but are not mentioned here. Their support and encouragement are deeply appreciated.

## Author contributions

**Conceptualization:** Khalid Alansari, Rafah Al Sayyed.

**Data curation:** Khalid Alansari, Rafah Al Sayyed.

**Funding acquisition:** Khalid Alansari.

**Investigation:** Khalid Alansari, Rafah Al Sayyed.

**Methodology:** Khalid Alansari, Rafah Al Sayyed.

**Project administration:** Khalid Alansari.

**Supervision:** Khalid Alansari.

**Validation:** Khalid Alansari, Rafah Al Sayyed.

**Writing** – **original draft:** Khalid Alansari.

**Writing** – **review & editing:** Khalid Alansari.
